# Ciprofloxacin-Induced Thrombotic Thrombocytopenic Purpura: A Case of Successful Treatment and Review of the Literature

**DOI:** 10.1155/2015/143832

**Published:** 2015-10-26

**Authors:** Hafiz Rizwan Talib Hashmi, Gilda Diaz-Fuentes, Preeti Jadhav, Misbahuddin Khaja

**Affiliations:** ^1^Division of Pulmonary and Critical Care Medicine, Bronx Lebanon Hospital Center, Bronx, NY 10457, USA; ^2^Department of Internal Medicine, Bronx Lebanon Hospital Center, Albert Einstein College of Medicine, Bronx, NY 10457, USA

## Abstract

A 49-year-old African American woman was admitted to our hospital with abdominal pain, nausea, vomiting, lethargy, and confusion. She was receiving ciprofloxacin for a urinary-tract infection prior to admission. Laboratory examination revealed anemia, thrombocytopenia, elevated lactate dehydrogenase, and serum creatinine. Peripheral smear showed numerous schistocytes, and the patient was diagnosed with thrombotic thrombocytopenic purpura (TTP). Ciprofloxacin was identified as the offending agent. The patient received treatment with steroids and plasmapheresis, which led to rapid clinical recovery. This is the first case to our knowledge of successfully treated ciprofloxacin-induced TTP; previously reported cases had fulminant outcomes. Quinolones are an important part of the antibiotic armamentarium, and this case can raise awareness of the association between quinolones and TTP. A high index of suspicion for detection and early and aggressive management are vitally important for a successful outcome.

## 1. Introduction

Ciprofloxacin is a DNA gyrase inhibitor that provides excellent coverage of gram-negative bacteria with marginal effectiveness against gram-positive bacteria. It is generally used to treat infections of bones and joints, endocarditis, gastroenteritis, malignant otitis externa, respiratory-tract infections, cellulitis, urinary-tract infections, prostatitis, anthrax, and chancroid. Common side effects include neurological symptoms (dizziness, insomnia, and nervousness), gastrointestinal symptoms (diarrhea, dyspepsia, and nausea), and transaminitis [1].

Quinolones should be prescribed with caution to patients with HIV infection because of the potential for associated hypersensitivity reaction [2]. Uncommon adverse effects of ciprofloxacin include renal failure, agranulocytosis, anemia, bone-marrow suppression, and thrombocytopenia. Although full manifestation of fluoroquinolone-induced thrombotic thrombocytopenic purpura (TTP) requiring plasmapheresis is extremely rare, there are documented cases of life-threatening complications of TTP due to moxifloxacin [3]. There is sparse literature on TTP caused by other fluoroquinolones including ciprofloxacin. Here, we report an unusual case of TTP associated with the use of ciprofloxacin that was successfully treated with steroids and plasmapheresis.

## 2. Case Presentation

A 49-year-old African American woman presented to the emergency room with complaints of nausea, vomiting, abdominal pain, and altered sensorium. The abdominal pain was poorly localized, dull, and associated with nonbloody, nonbilious vomiting. The patient reported subjective fever and chills. Her medical history was remarkable for hypertension controlled with diet and lifestyle modifications. She was seen in another hospital 5 days earlier for urinary-tract infection and was prescribed ciprofloxacin and discharged. The patient denied taking any other prescribed or nonprescribed medications. She was a light smoker and denied any history of illicit or recreational drug use. On physical examination, she was in mild distress and confused but had normal vitals, and neurological examination did not reveal sensory or motor deficit. Cardiorespiratory examination was unremarkable. Abdominal examination revealed minimal diffuse tenderness without guarding, rigidity, or other signs of peritonitis. Bowel sounds were present, and no visceromegaly was appreciated. Skin examination did not reveal any petechiae or rash.

**Figure 1:**
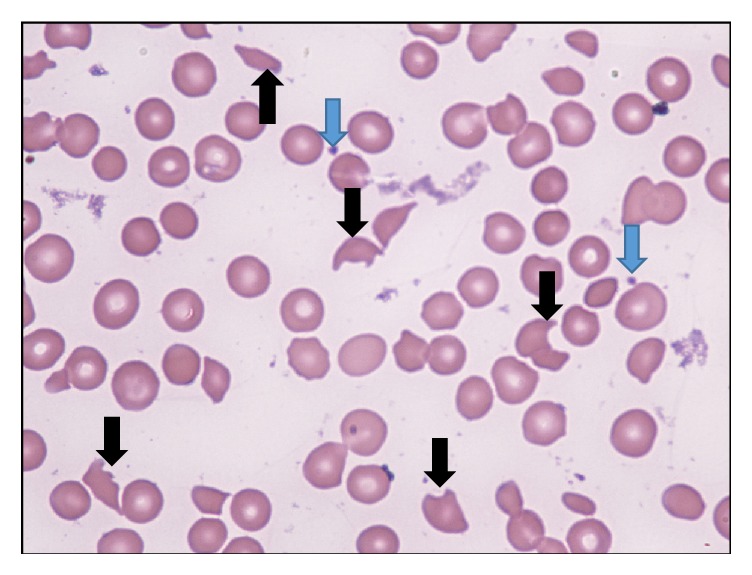
Peripheral smear shows moderate to severe schistocytes (black arrows) and very few platelets (blue arrows).

Laboratory examination revealed anemia (hemoglobin: 8.1 g/dL, Ref.: 12–16 g/dL) and thrombocytopenia (platelets: 13 k/*μ*L, Ref.: 150–450 k/*μ*L) with 1959 U/L lactate dehydrogenase (Ref.: 100–190 U/L). Serum haptoglobin was 10 mg/dL (Ref.: 30–200 mg/dL), and the patient had reticulocyte count of 14%. Peripheral smear revealed moderate to severe schistocytosis and few platelets (Figure 1). Serum creatinine was 1.3 mg/dL with a baseline of 0.8 mg/dL (Ref.: 0.5–1.5 mg/dL). Serum lipase level was normal. Chest roentgenogram and computed tomography of the brain and abdomen were unremarkable. A clinical diagnosis of TTP was made, and the patient was admitted to the medical intensive care unit.

**Figure 2:**
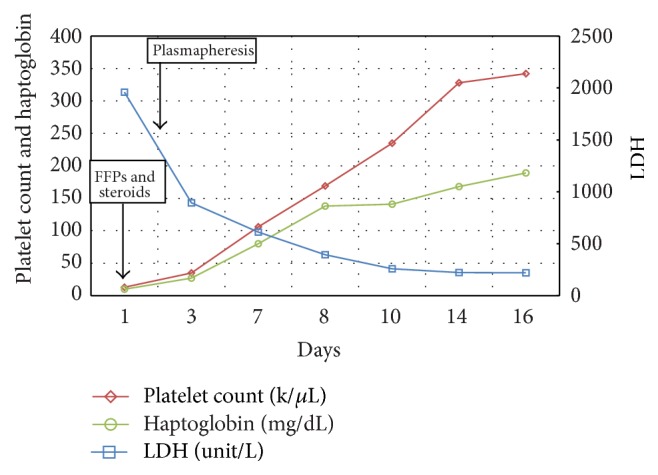
Platelet count, haptoglobin, and LDH over the course of treatment.

Coomb's test, HIV antibody test, hepatitis panel, and septic work-up including blood, urine, and stool were negative. The patient's ADAMTS 13 activity was <3 (Ref.: 68–163). During hospitalization, the patient received fresh frozen plasma while awaiting plasmapheresis. Eight sessions of plasmapheresis with one volume exchange in each session were done. In addition, patient received intravenous methylprednisolone 62.5 mg two doses and 40 mg of intravenous methylprednisolone twice a day for two weeks (until a sustained rise in platelet was seen), followed by a gradual taper with oral prednisone. During the course of treatment, the hematological abnormalities, that is, platelets, LDH, and haptoglobin, returned to normal (Figure 2). The patient was discharged and remained asymptomatic with stable hematological profile during follow-up in the ambulatory clinic.

## 3. Discussion

TTP, first described by Moschcowitz in 1924 [4], is a form of thrombotic microangiopathy characterized by systemic microvascular platelet aggregation and erythrocyte destruction. It has an estimated annual incidence in the United States of 4 to 11 cases per million people and is more common in women and individuals of African descent [5]. TTP is associated with a pentad of clinical signs and symptoms including thrombocytopenia, microangiopathic hemolytic anemia, neurological abnormalities, renal failure, and fever. Since a randomized control trial demonstrated the efficacy of plasma exchange therapy in the treatment of TTP [6], microangiopathic hemolytic anemia and thrombocytopenia without any alternate etiology have been considered sufficient for diagnosis [7]. The use of those criteria for diagnosis has resulted in a 7-fold increase in the number of patients treated for TTP [8].

TTP has diverse etiologies including infections, medications, and idiopathic and familial causes. Drug-associated TTP represents about 12% of all cases [9]. Before the advent of plasma therapy, most of the patients presenting with acute TTP died: the case-fatality rate reported in clinical series was near 100% until the 1960s [10, 11].

A test for severe deficiency (activity < 5) of von Willebrand factor- (vWF-) cleaving protease called ADAMTS 13 is 100% sensitive and specific for the diagnosis of TTP. ADMATS 13 cleaves vWF multimers, and its absence results in large vWF multimers that react with platelets, resulting in the widespread formation of platelet thrombi, which are responsible for the clinical presentation of TTP [12]. Although it is not always detected, the presence of ADAMTS 13 inhibitor suggests the acquired forms of TTP. Congenital TTP usually presents in early childhood in individuals with a positive family history of similar disorders.

For adults, plasma exchange is the only treatment supported by well-founded data. The effectiveness of plasma exchange is attributed to the removal of ADAMS 13 autoantibodies and the restoration of ADAMTS 13 activity [4, 13]. Plasma exchange is effective, however, even in patients who do not have severe ADAMTS 13 deficiency [14].

Current guidelines recommend daily plasma exchange with replacement of 1.0 to 1.5 times the predicted plasma volume of the patient [15, 16]. Further recommendations include the use of glucocorticoids in all patients with TTP and continued plasma exchange therapy for at least 2 days after the platelet count returns to normal [16, 17].

Many commonly prescribed drugs and pharmacological substances have been associated with the development of TTP. It is difficult to evaluate case reports describing associations between various drugs and the disease entity, however, because there are no standardized criteria to document the drugs as the probable cause of TTP. The incidence of TTP remains poorly defined and is dependent on voluntary reporting. Moreover, the case reports of drug-induced TTP have not been reviewed systemically to determine a formal cause-effect relation, as has been done for drug-induced thrombocytopenia [18].

Fluoroquinolones are an emerging but underrecognized cause of drug-induced thrombocytopenia. Cheah et al. described fluoroquinolone-induced thrombocytopenia [19]. Here, we focus on fluoroquinolones as a cause of TTP. Fluoroquinolones have a wide range of immune-hematopathologic effects, and the exact mechanisms for most of those effects remain unclear. It has been hypothesized that similarities in the chemical structures of quinolones and quinine might be contributory [19], because quinines are a well-recognized cause of thrombocytopenia [20].

Fluoroquinolones have been associated with TTP and the hemolytic-uremic syndrome. One of the largest published series described “temafloxacin syndrome” in which 95 patients developed hemolytic anemia, thrombocytopenia, and renal failure following treatment with temafloxacin, which was subsequently withdrawn from the market [21]. A comprehensive review of the existing literature on immune thrombocytopenia and TTP secondary to drugs did not indicate fluoroquinolones as a causative agent [7, 22, 23]. The few previous reports of ciprofloxacin-induced TTP described a fulminant course with very high mortality. Mouraux et al. described a case of a 43-year-old female who developed fulminant TTP early in a course of ciprofloxacin therapy. That patient was initially suspected to have meningoencephalitis because of marked thrombocytopenia, however, and spinal tap was delayed. Later on, cerebrospinal fluid analysis was unremarkable, and the patient was diagnosed with TTP; however, the patient died due to systemic complications [24]. Tuccori et al. described a case of TTP in a 30-year-old female receiving ciprofloxacin treatment for a urinary-tract infection, who died within 17 hours of diagnosis [25].

## 4. Conclusion

Ciprofloxacin is a common and widely used antibiotic with excellent tissue penetration and gastrointestinal absorption. Our patient highlighted a rare but, if unrecognized, potentially fatal complication that clinicians should be aware of. There are no established risk factors to prospectively determine which patients are at increased risk of developing TTP due to ciprofloxacin. Therefore, early recognition and aggressive management are of paramount importance for survival.
